# Utilization of Brick Powder in Blended Cement Compositions: Rheological, Mechanical, and Microstructural Properties

**DOI:** 10.3390/ma18225120

**Published:** 2025-11-11

**Authors:** Vitalii Kryzhanovskyi, Jeanette Orlowsky, Jan Skocek, Marina Macias Barrientos

**Affiliations:** 1 Faculty of Architecture and Civil Engineering, TU Dortmund University, 44227 Dortmund, Germany; jeanette.orlowsky@tu-dortmund.de; 2 HeidelbergCement AG, Global R&D, Oberklamweg 2-4, 69181 Leimen, Germany; jan.skocek@heidelbergcement.com; 3 Faculty of Mechanical Engineering, TU Dortmund University, 44227 Dortmund, Germany; marzwill@gmail.com

**Keywords:** brick powder, pozzolanic activity, strength activity index, microstructure, C-S-H/C-A-S-H phases, slag cement, sustainable binders

## Abstract

The growing demand for eco-efficient construction materials has driven the development of low-clinker cement systems incorporating recycled mineral additives. Finely ground brick powder represents one of such materials with high pozzolanic potential. This article presents an experimental study on the effect of partially replacing slag cement CEM III and ordinary rapid-hardening cement CEM I with brick powder waste of different chemical compositions and fineness levels (63, 32, and 15 µm) on the physical and mechanical properties of blended cement mortars. Compressive and flexural strengths were determined at 2, 7, and 28 days, along with the strength activity index (SAI). Additionally, the setting times and standard consistency were investigated, with the latter showing a correlation with the workability of fresh mortars. Comprehensive microstructural analysis (TGA, SEM, EDX) confirmed the pozzolanic activity of the brick powder, which was manifested by the formation of C-S-H and C-A-S-H phases. The highest strength characteristics were achieved with a 15% replacement of cement by brick powder with a fineness of 32 μm and an increased SiO_2_ content (63.06%). Comparative analysis with fly ash- and silica fume-modified mortars revealed that brick powder exhibits comparable performance, confirming its suitability as an active mineral additive.

## 1. State of the Art

Cement is still the key binding material for the construction industry. The basis for its production is a mixture of clay and natural limestone, which is fired at a temperature of 1450 °C and then ground in special mills. This technological process has a significant environmental impact. Thus, depending on the type of cement, around 300–800 kg of CO_2_ can be emitted per ton of material produced [1,2,3,4,5]. In light of dwindling clay and limestone resources and the rising energy costs associated with clinker manufacturing (particularly for burning and grinding), the need for alternative solutions has become economically and environmentally imperative for the cement and concrete production industry.

One of the effective solutions is the use of supplementary cementitious materials (SCMs) with pozzolanic properties [6,7,8]. The effectiveness of these materials lies in their amorphous SiO_2_, Al_2_O_3_, and Fe_2_O_3_ content, which reacts with free Ca(OH)_2_ to form additional C-(A)-S-H and AFm phases during cement hydration [9,10,11]. This leads to a denser cementitious matrix resulting in improved physical and mechanical properties of pozzolan-modified cement composites and derived concretes [12,13,14,15,16,17].

In contemporary practice, several pozzolanic materials are extensively utilized: silica fume [18,19,20], fly ash [21,22], calcined clays [23,24,25], blast-furnace slag [26,27,28] zeolite tuff [29,30,31], and rice husk ash [32,33,34]. Beyond conventional pozzolans, the construction sector is increasingly turning to demolition waste powders (DWPs) as innovative, eco-friendly alternatives. By utilizing DWP-derived materials such as brick powder (BP), concrete powder, or their blends [35,36,37], the industry can significantly mitigate environmental harm, curb global greenhouse gas emissions, and advance circular economy principles in sustainable construction. In recent years, BP has emerged as a focal point for research and application, with the cement and concrete sectors increasingly adopting it as a viable SCM [38,39,40,41,42]. This shift underscores a broader commitment to eco-efficient practices, leveraging waste-derived resources to reduce reliance on conventional clinker while maintaining material performance.

Experiment [43] demonstrates that increasing the specific surface area of BP from 0.578 m^2^/g to 1.69 m^2^/g leads to a significant increase in compressive strength of mortar at 7 days (W/C ratio 0.5), exceeding 12.3% and reaching 42.4 MPa. Mortar flexural strength also saw an increase of over 19.6%, reaching 4.3 MPa. In contrast, at 28 days of curing, the compressive strength rises in samples with finer BP fractions not exceeding 3% (45.6 MPa), and the flexural strength growing no more than 2.6% (4.8 MPa). These results were achieved with a 20% replacement of CEM II/A-V 42.5 cement. A similar trend is observed when replacing 30% of cement with BP having a specific surface area in the range of 0.253 m^2^/g to 0.795 m^2^/g [44]. At 7 and 28 days of mortar hardening (W/C ratio 0.5), the compressive strength of cement samples based on CEM II 42.5 with finer BP was comparable to specimens containing coarser ground BP fraction. Both studies note an increase in the water demand of cement pastes and mortars with an expanded amount of BP. However, the higher fineness of the BP helps mitigate this effect. Additionally, a smaller particle size leads to shorter initial and final setting times. The same behavior of cement pastes with a W/C ratio 0.4 is remarked when using 30% recycled BP for ordinary cement CEM I 42.5 substitution [45]. The compressive strength values at 3, 7, and 28 days of curing were 28 MPa, 42.4 MPa, and 45.2 MPa, respectively. These values were 35.7%, 12%, and 12.8% lower compared to the reference specimens. A comparison of data on the use of 20% BP as a replacement for CEM I 42.5 in cement–sand mortars (W/C ratio 0.5) shows an improvement in strength characteristics when the average particle size is reduced from 60 to 40 µm [46]. At the age of 7 days, the compressive strength of the specimens was 40 MPa, and the flexural strength was 6.8 MPa. These values were 12.5% and 5.9% lower than the reference samples. However, at the age of 28 days, these properties reached the level of the control specimens: 50 MPa for compressive strength and 8 MPa for flexural strength. For its part, the results from [47] show a positive trend in mortar strength development (W/C ratio 0.5) when 15% of cement clinker is replaced with BP waste with a reduction in particle size from 42 µm to 6 µm. The early compressive strength of the samples at 3 days was only 1.9% lower than the baseline specimens. By 28 days, however, this parameter exceeded the control mixture by 5.7%, reaching 44.5 MPa. Increasing the content of recycled BP to 30% proved inefficient regardless of the average particle size, as it led to a significant strength reduction of 10.7–28%. Moreover, the study highlights a decline in workability of the mixtures with higher BP amount. A comprehensive study by [48] investigated the partial replacement of CEM I 42.5 with 25% BP of varying fineness in cement–sand mortars with a W/C ratio 0.5. The analysis included 26 batches of samples with this additive, differing in genesis, age, and production method. The study found that the effectiveness of BP as a pozzolanic additive depends on its CaO and MgO content. Additionally, powders with high SiO_2_ content can also exhibit significant reactivity, with their efficiency improving as fineness increases. At the same time, [45] emphasizes the crucial role of hydrate morphology in the interaction between BP and cement, which significantly affects the adhesion of newly formed nucleation phases. Microstructural analysis confirms the pozzolanic activity of BP, especially at higher degrees of fineness [47].

Mortar compositions based on rapid-hardening cement CEM I 42.5 R/SR show the highest compressive strength of 62 MPa and flexural strength of 4.9 MPa at 28 days (W/C ratio 0.5) when replacing the binder with 35% BP [49]. On the other hand, data from [50] show that the effective dosage of this additive in combination with CEM I 42.5 R is limited to just 10%. Cement pastes (W/C ratio 0.37–0.43) prepared with CEM I 52.5 and a substitution of 10–20% BP waste with an average diameter of 16 µm exhibited optimal compressive strength at both early and design ages. Depending on the specific composition, the characteristic compressive strength ranged from 59 to 63 MPa, and the 3-day compressive strength was no less than 25 MPa, which was comparable to samples without the BP. A denser microstructure in the blended cement pastes was observed due to the pozzolanic reaction [37].

Particular interest lies in blended cement–sand mortar compositions incorporating blast-furnace slag and BP. Data from [51] indicate that even if a low dosage of BP (up to 10%) with an average diameter of 21 µm is used to replace CEM I 42.5 N cement, a reduction in binder activity is recorded (W/C ratio 0.5). This is evident in a 28% average lower early compressive and flexural strength at 2 days, and a 40% average lower final strength at 28 days. However, a combination of 50% cement, 10% BP, and 40% slag showed improved results at 28 days of curing. The compressive strength was 40.8 MPa compared to 37.5 MPa for the composition with 10% BP replacement. Flexural strength was 7.4 MPa for the slag composition, compared to 6.7 MPa when using only 10% BP. Despite these later gains, the early flexural strength of the slag-containing mixture at 2 days was 2.2 MPa, which was two times lower than specimens with BP. Compressive strength for the slag mixture was 8.4 MPa, 2.3 times lower than the BP samples at the same early age. The use of 30% slag was more effective than 30% BP in terms of early and ultimate strength development in blended cement pastes (W/C ratio 0.5) based on CEM II 42.5 [52]. Furthermore, the introduction of modifiers had a lesser effect on changes in compressive strength than on flexural strength. Microstructural analysis indicates that BP requires less portlandite than blast-furnace slag for the formation of additional C-(A)-S-H phases. Four types of C-(A)-S-H morphology are observed in the blended cement paste with slag and BP, suggesting continuous hydration in this type of composite.

To ensure methodological consistency, the literature review was limited to compositions that did not contain plasticizers, setting accelerators, alkali activators, expansive agents, or other chemical modifiers, in order to allow a clear comparison of the brick powder effect. The analyzed scientific sources reveal ambiguous results regarding the impact of replacing cement with BP waste. Experimental groups emphasize the importance of the phase composition of the BP by-product, as well as its particle size. Despite the diverse findings on the mechanical strength and microstructure of mortars with BP, the majority of data has been obtained using CEM I and CEM II. Consequently, there is a significant unexplored area concerning the effectiveness of BP as a mineral additive in composites based on slag cement CEM III, which is promising due to its lower CO_2_ emissions during production. Additionally, it is worth noting that the properties of cementitious composites modified with BP using rapid-hardening CEM I are insufficiently studied. The authors of this research aimed to investigate the influence of BP with varying chemical compositions and fineness on the rheological, strength, and microstructural characteristics of blended cement composites based on CEM III and rapid-hardening CEM I.

## 2. Materials and Experimental Program

### 2.1. Raw Materials and Mix Proportions

Two types of cement produced by Heidelberg Cement AG (Heidelberg, Germany) [53] slag Portland cement CEM III/A 42.5 N and rapid-hardening Portland cement CEM I 42.5 R were used for the preparation of cement–sand mixtures. Mineral additives were used—red brick powder waste (BPL) from Lücking GmbH & Co. (Warburg, Germany) with average particle sizes of 63, 32, and 15 µm and red brick powder waste (BPLB) from Leipfinger-Bader GmbH (Vatersdorf, Germany) with average particle sizes of 63 and 32 µm. The BPL and BPLB powders were obtained from waste clay bricks and shale bricks, respectively. Both types of bricks were originally manufactured through conventional ceramic processing, involving extrusion, drying, and firing at approximately 900–1000 °C. Quartz sand with a fineness modulus of 2.9 was used as fine aggregate. Tap water was used to mix the investigated compositions.

Based on literature sources, replacement levels of 15% and 30% by weight of cement were selected for BP. To evaluate the effectiveness of BP, blended cement–sand mixtures were also produced with 15% and 30% fly ash (FA), and silica fume (SF) at 7.5% and 15%. The chemical composition of the binders and mineral additives is presented in Table 1. The compositions of the investigated mixtures are given in Table 2. All studied mortars were prepared with a W/C ratio of 0.5 and a 1:3 binder-to-sand ratio.

The data in Table 1 indicate that the sum of oxides SiO_2_ + Al_2_O_3_ + Fe_2_O_3_ is 79.85% for BPL and 84.78% for BPLB. Thus, according to the requirements of [55,56] (minimum sum of SiO_2_ + Al_2_O_3_ + Fe_2_O_3_ = 70%), the brick powder wastes used in this study classified as SCM.

### 2.2. Standard Consistency and Setting Time

**Figure 1:**
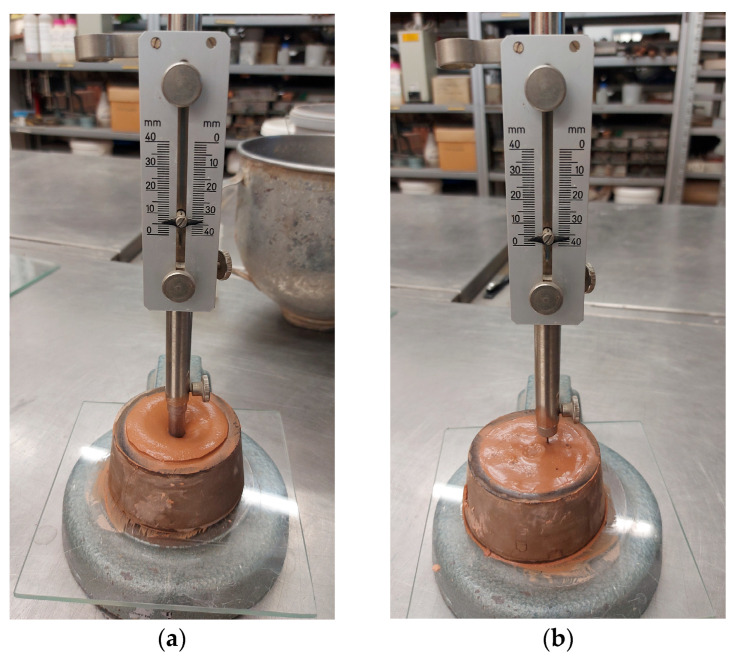
Standard consistency test (**a**), setting time test (**b**).

According to the method [57], the standard consistency, initial, and final setting times were determined for the all studied blended cement paste compositions (Figure 1).

### 2.3. Workability

**Figure 2:**
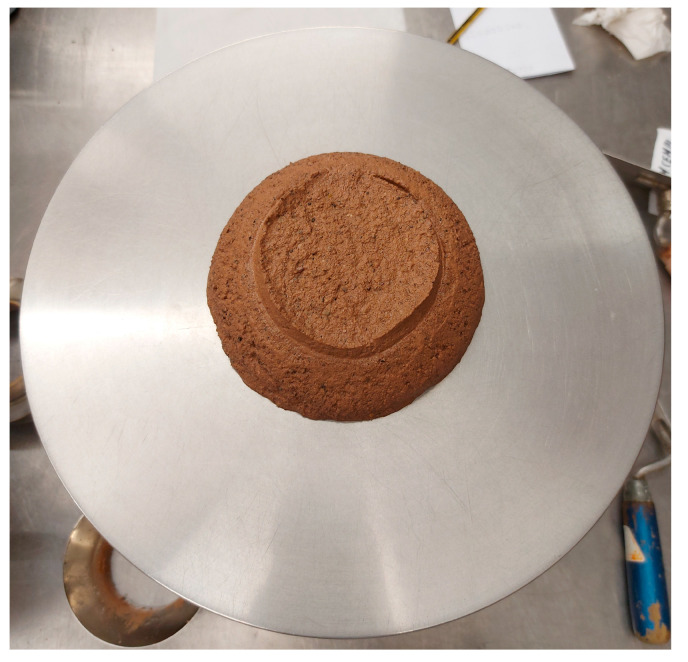
The flow spread diameter of the mixture after the workability test.

The mixtures were prepared in accordance with [58] standard methodology. Then, the standard cone was filled in two layers and slowly lifted vertically. Subsequently, the mixture was jolted 15 times. The spread of the mixture was measured in two perpendicular directions, and the average value was calculated to determine the consistency of the tested mixtures. The workability test is illustrated in Figure 2.

### 2.4. Water Absorption of Mineral Additives

The water absorption of the mineral additives (BP, FA, and SF) was determined by mass according to a gravimetric method. For each additive, a 100 g sample was oven-dried at 105 ± 5 °C for 24 h until constant mass (*m*_1_) was achieved. The dried sample was then immersed in 500 g of water and kept under static conditions for 48 h. After soaking, the excess surface water was carefully removed, and the saturated sample mass (*m*_2_) was recorded. The water absorption by mass (*W_m_*) was calculated according to Equation (1):

(1) $$W_{m}=\frac{m_{2}-m_{1}}{m_{1}}\cdot 100\%,$$

### 2.5. Compressive and Flexural Strength

For each mixture composition listed in Table 1, three batches of 4 × 4 × 16 cm specimens were manufactured to determine the compressive and flexural strength at the age of 2, 7, and 28 days. Depending on the composition of the investigated mixtures, cement, sand, and mineral additives were mixed until homogeneous. Then, mixing water was added and three specimens for each batch were produced. The molds were filled in two layers and then compacted on a vibrating table according to [58]. During the initial 24 h, the specimens were cured in a climatic chamber at 20 °C and 90% relative humidity. Then the specimens were demolded and stored in a bath with water temperature of 20 °C. On the day of testing, the specimens were first removed from the bath, wiped with a dry towel and kept in the test room at a temperature of 20 °C and relative humidity of 60% for 15 min prior to strength testing.

### 2.6. Strength Activity Index (SAI)

The SAI allows the determination of the efficiency of mineral additives in the investigated blended cement mixture compositions from the point of view of pozzolanic properties. This is a simple technique that allows the evaluation of a material for its reactivity based on its strength characteristics. According to the standard methods [55,56,59,60] the SAI was determined using Equation (2) for samples at the age of 7 and 28 days.

(2) $$SAI=\frac{f_{c1}}{f_{c2}}\cdot 100\%$$

where

*f_c_*_1_—compressive strength of mortar samples with pozzolanic additive;

*f_c_*_2_—compressive strength of mortar samples without pozzolanic additive.

### 2.7. Microstructure Formation Analysis

#### 2.7.1. Thermogravimetric Analysis (TGA)

To evaluate the pozzolanic activity of the investigated mineral additives in blended cement compositions, as well as to perform a quantitative analysis of the hydrate phases, TGA analysis was conducted [61]. Following mechanical strength testing, the specimens were manually crushed using a hammer and sieved through a 63 µm mesh. A 50 mg sample of the resulting powder was precisely weighed, transferred to an aluminum crucible, and TGA analysis was performed under the following conditions: temperature range of 40–1000 °C (heating rate: 20 °C/min), N_2_ gas flow rate of 40 mL/min. The hydrated blended cement powders were studied at the age of 90 days.

#### 2.7.2. Scanning Electron Microscopy Analysis (SEM)

SEM was performed to visually analyze microstructural features of blended cement powders. Hydrated cement powder (previously used for TGA analysis) was mounted on aluminum stubs using double-sided carbon tape. To prevent charge accumulation, a thin carbon coating was applied using vacuum sputtering. SEM imaging was carried out using a Zeiss Crossbeam XB 550L scanning electron microscope (Oberkochen, Germany).

#### 2.7.3. Energy-Dispersive X-Ray Spectroscopy Analysis (EDX)

To verify the phase composition and assess the distribution of mineral additives, EDX analysis was performed in parallel with SEM analysis using an Oxford Instruments (Wiesbaden, Germany) detector at an accelerating voltage of 10 kV to minimize damage to the hydrate phases. Three spectra were analyzed for each sample. Based on the data obtained, a correlation with the TGA and SEM results was conducted.

## 3. Test Results and Analysis

### 3.1. Standard Consistency and Setting Time

**Figure 3:**
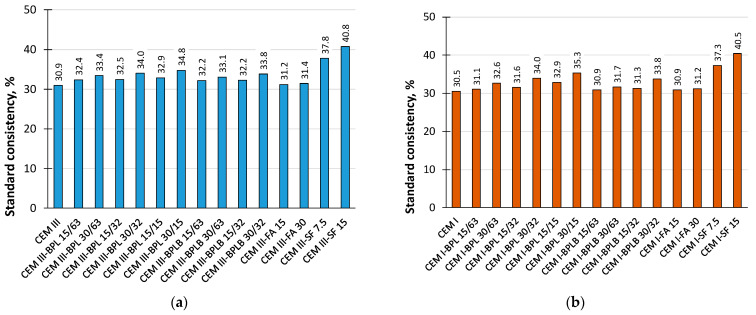
Standard consistency of the studied blended cement pastes based on CEM III (**a**), and based on CEM I (**b**).

Figure 3 reflects the effect of BP, FA, and SF on the standard consistency of blended cement pastes based on CEM III and CEM I. As shown in Figure 3, the water demand increases with a higher BP replacement level. This effect is more pronounced in CEM III-based pastes. At 30% replacement, the impact of BP fieness on standard consistency is more substantial than at 15%, regardless of thebinder type. Overall, the water demand of BP-blended pastes is only marginally higher than that of FA mixtures and signifiantly lower than those containing SF.

**Figure 4:**
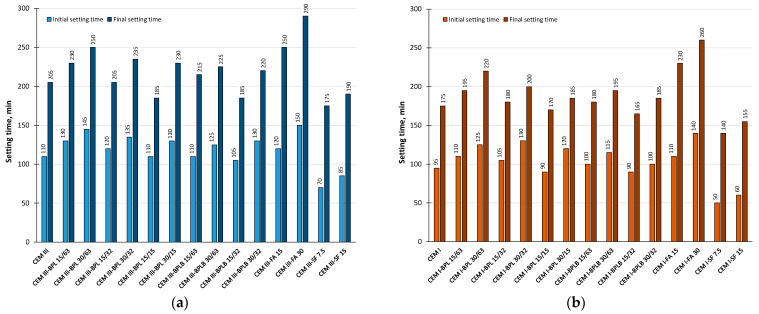
Initial and final setting times of the investigated blended cement pastes based on CEM III (**a**), and based on CEM I (**b**).

Figure 4 reflects the inflence of BP, FA, and SF on the initial and finals setting times of blended cement pastes.

Analysis of the data in Figure 4 shows that the initial and final setting times of the cement pastes lengthened with increasing BP replacement level, regardless of the cement type or BP fineness. However, when the fineness of BPL was increased to 15 µm at a 15% substitution level, the setting times became comparable to the control samples for both cement types. It is also noteworthy that the modification of cement pastes with BPLB with a fineness of 32 µm, at a quantity of 15%, led to similar setting time results. This acceleration effect is attributed to the smaller particle diameter of BP, which provides more crystallization and nucleation sites, thereby accelerating hydration in blended cement pastes. Despite its coarser grind, the enhanced performance of BPLB is linked to the chemical composition, namely a higher SiO_2_ content than that of BPL. These findings are consistent with existing research [38,62]. The longer setting times of FA-modified pastes result from its slower hydration kinetics compared to SF and BP, which aligns with the studies [63,64,65]. Thus, all BP-blended cement pastes meet the requirements of standard [53], specifying a minimum initial setting time of 60 min for Class 42.5 cements.

### 3.2. Workability

**Figure 5:**
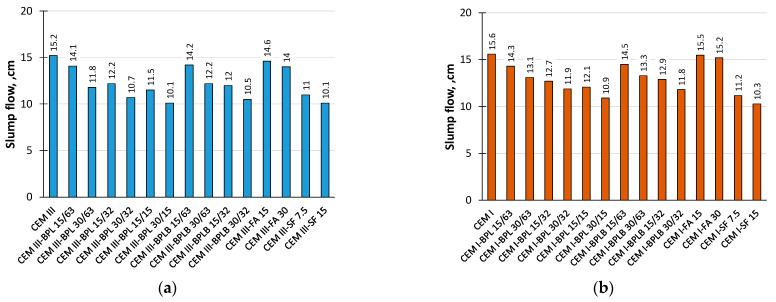
Workability of the studied modified cement–sand mortars based on CEM III (**a**), and based on CEM I (**b**).

**Figure 6:**
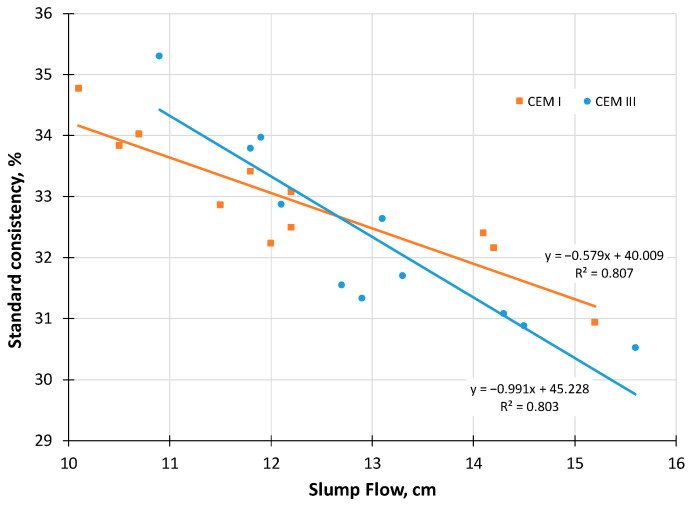
Correlation dependencies between the standard consistency of blended cement pastes with BP and the workability of cement–sand mortars based on them.

The workability results of the investigated blended cement–sand mortar compositions (Table 2) are presented in Figure 5. Under an equal W/C ratio, cement–sand mortars modified with FA demonstrated the highest workability. This is attributed to the morphological characteristics of FA particles, which are more spherical and rounded as confirmed in studies [66,67]. Increasing the dosage of BP for partial cement replacement and improving its fineness reduces the workability of the tested mortars, regardless of whether CEM III or CEM I binder is used. The workability results show a strong correlation with the water demand of blended cement pastes at standard consistency (Figure 6).

### 3.3. Compressive and Flexural Strength

**Figure 7:**
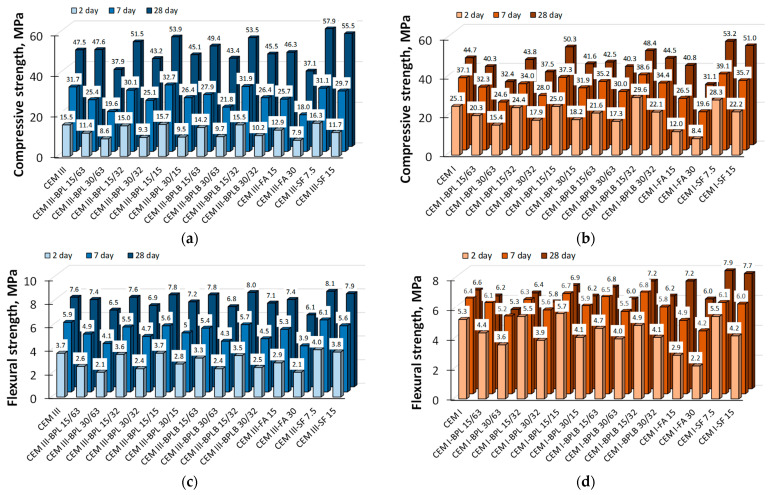
Strength properties of the investigated mortars: compressive strength on CEM III (**a**), compressive strength on CEM I (**b**), flexural strength on CEM III (**c**), flexural strength on CEM I (**d**).

The diagrams in Figure 7 illustrate the effect of blended cements on the compressive and flexural strength of the studied cement–sand mortars. The densities of all investigated mortar samples were found to range between 2210 and 2240 kg/m^3^.

The highest compressive strength at all test ages (2, 7, and 28 days) for both cement types was achieved with a 15% replacement of BPLB (32 µm). For CEM III mortars, the 2- and 7-day strength was comparable to the control (15.5 and 31.9 MPa, respectively), while the 28-day strength exceeded the control by 12.6%, reaching 53.5 MPa. For CEM I mortars, strength values of 29.6, 38.6, and 48.4 MPa at 2, 7, and 28 days exceeded the control by 17.9%, 4%, and 8.3%, respectively. The flexural strength of CEM III mortars was 5.7% and 3.5% lower than the reference at 2 and 7 days (3.5 and 5.7 MPa), but 5.3% higher at 28 days (8.0 MPa). For CEM I mortars, early (2-day) flexural strength was 8.2% lower (4.9 MPa), but later values (6.8 and 7.2 MPa at 7 and 28 days) exceeded the reference samples by 6.3% and 9.1%. This can be explained by the cumulatively higher content of SiO_2_, Al_2_O_3_, Fe_2_O_3_ oxides (Table 1) contributing to the development of a denser cement stone structure due to the formation of additional C-(A)-S-H phases, which is confirmed by researchers [48,68]. Consequently, growing the fineness of BLB could not compensate for the deficiency in SiO_2_, Al_2_O_3_, Fe_2_O_3_ in the investigated blended cement–sand compositions. For this reason, the strength of mortars using the secondary product BLB was in all cases lower than the strength of mortars using BLBP. It should be noted that the strength characteristics of blended cement–sand mortars of a specific composition in combination with BP waste are on par with compositions that include SF or FA.

**Figure 8:**
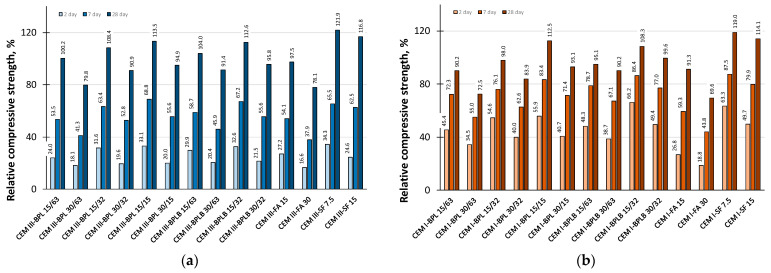

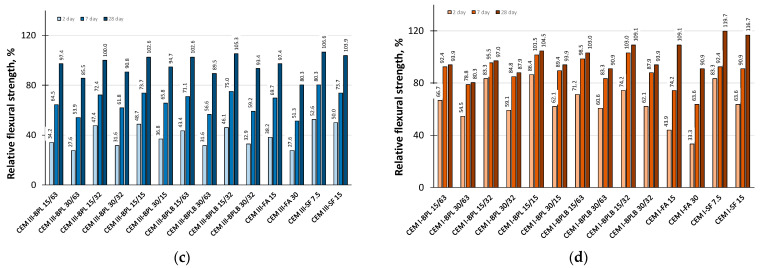
Strength development of modified mixtures relative to the 28-day strength of the control mixture: compressive strength on CEM III (**a**), compressive strength on CEM I (**b**), flexural strength on CEM III (**c**), flexural strength on CEM I (**d**).

The increased fineness of BP enhances the compressive and flexural strength gain compared to mixtures with FA and is comparable to mixtures containing SF, as shown in the diagrams in Figure 8. This effect is more pronounced for CEM I-based compositions compared to CEM III-based compositions at early hardening stages (2–7 days). In turn, at the design age of 28 days, CEM III-based mortars show higher strength gain compared to CEM I-based mortars. This behavior is caused by the synergistic effect of structure formation in modified slag systems [69,70]. The nature of flexural strength development in mixed compositions with CEM III (Figure 8c) is close to the corresponding compressive strength values. In contrast, for most CEM I-based compositions, there is a tendency for a more intensive strength gain at 2 days for compositions containing BP, while the difference in flexural strength development between 7 and 28 days of hardening is minimal. Meanwhile, CEM I-based compositions with FA and SF demonstrate a similar trend in flexural strength gain as in compression [71,72,73]. The higher early-age flexural strength of CEM I-based compositions containing BP can be explained by the synergy between the morphology of the additive and the kinetics of the binder hydration. BP particles, characterized by an angular shape unlike the spherical particles of FA and the ultra-dispersed SF, create a developed structure of mechanical contacts in the cement matrix. This micro-reinforcing effectively inhibits the development of microcracks. Simultaneously, the intense early hydration of CEM I ensures the rapid formation of a strong crystalline framework, which firmly bonds with the angular particles, integrating them into the composite structure. As a result, a more homogeneous and structurally sound matrix is formed, which is manifested in the higher flexural strength values compared to compositions based on the slower-hydrating CEM III.

The improved consistency of the obtained experimental strength data can be attributed to the water absorption by the mineral additives used, as presented in Table 3. The increased dispersity of BP promotes rapid strength development in mortars by binding a significant amount of water. This process lowers the effective water-to-cement ratio, thereby enhancing early-age strength (2–7 days). At a later curing stage (7–28 days), the water begins to be released, providing the necessary moisture for the pozzolanic reaction to proceed.

### 3.4. Strength Activity Index (SAI)

**Figure 9:**
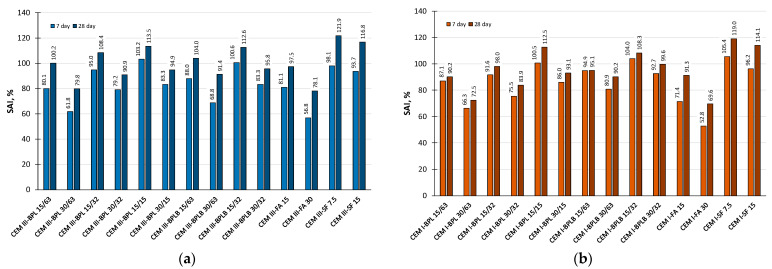
SAI of the investigated mineral additives in blended cement systems based on CEM III (**a**), and based on CEM I (**b**).

The SAI determines the reactivity of different types of additives and therefore the effectiveness of their use at different hardening ages. According to the standards [55,56,59,60], the materials used can be classified as active mineral additives if the SAI at ages of 7 and 28 days exceeds 75% in relation to the control samples. The SAI data are presented in Figure 9.

According to the experimental data obtained, all materials, except for BP in a 30% binder replacement quantity with a fineness of 63 µm, and FA in a 30% quantity, can be classified as active mineral additives in combination with CEM III. Since the SAI is directly related to compressive strength, optimal results were achieved with BPL 15/15 and BPLB 15/32 products. Concurrently, when using CEM I, from an SAI perspective, various modifications are possible with the exception of 30% BPL with a fineness of 63 µm and all FA dosages.

### 3.5. TGA

**Figure 10:**
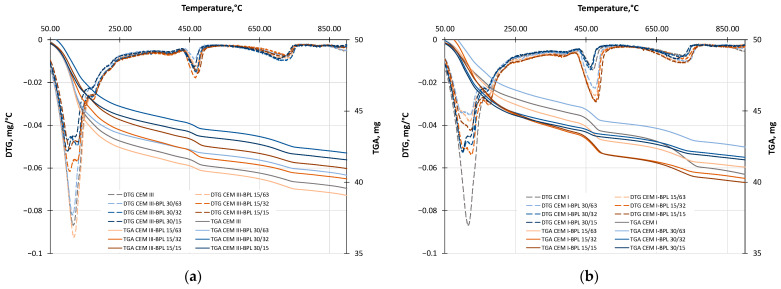
TGA and DTG curves of the investigated blended cement mortars based on CEM III with BPL (**a**), and based on CEM I with BPL (**b**).

The TGA data are shown in Figure 10 (solid lines). The TGA curves were differentiated to obtain the derivative thermogravimetry (DTG) curves (dashed lines), which are also displayed in Figure 10.

**Figure A1:**
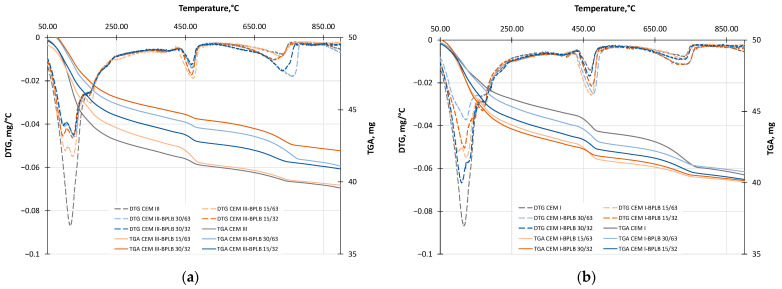

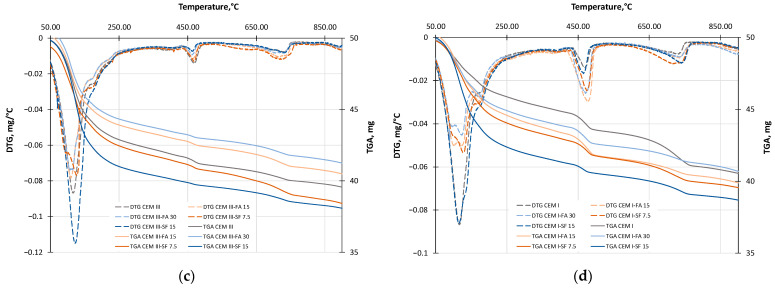
TGA and DTG curves of the investigated blended cement mortars based on CEM III with BPLB (**a**), based on CEM I with BPLB (**b**), based on CEM III with FA and SF (**c**), and based on CEM I with FA and SF (**d**).

Analysis of the results in Figure 10a demonstrates that reducing the particle size of BPL from 63 µm to 15 µm does not lead to an increase in the intensity of C-S-H phase formation (as evidenced by the comparison of DTG peaks in the temperature range of 120–150 °C). In contrast, the compositions with increased BPL fineness (32 µm and 15 µm) are characterized by two asymmetric maxima, unlike the samples containing BPL with a median diameter of 63 µm. This may indicate the formation of a more complex polymerized C-(A)-S-H structure [74,75]. The most optimal replacement for CEM III using BPL is 15% with a fineness of 15 µm. This is confirmed by its highest strength among all compositions based on CEM III with BPL. Active portlandite consumption is also observed in this modified system (reduction in the peak in the range of 400–500 °C), which confirms the pozzolanic reaction. Despite the high pozzolanic activity of BPL at 30% dosages, the reduction in strength may occur due to increased particle agglomeration and, as a result, the presence of microstructural defects [76]. All investigated samples with the by-product BPL are characterized by moderate carbonation, regardless of the powder’s fineness and its amount, as evidenced by nearly identical peaks in the 600–800 °C range. When modifying cement pastes with BPLB (Figure A1a), similarly shaped and intense C-S-H dehydration peaks are observed. This also indicates a greater variety of hydrate phases, even with a BPLB fineness of 63 µm. This result may be due to the increased SiO_2_ content in BPLB compared to BPL. Despite the moderate pozzolanic activity of BPLB at 15% and a fineness of 32 µm for replacing CEM III, the high strength of the powder-modified cement mortars is confirmed (Figure 7a,c). Although there is high pozzolanic activity in the 30% BPLB with a fineness of 32 µm (as evidenced by the highest degree of portlandite consumption), the lower strength of these samples is likely due to the formation of a large quantity of particle agglomerates. This leads to an increase in the porosity of the cement matrix [77], and despite the favorable chemical composition (high SiO_2_ content), the physical structure of the particles ultimately governs the strength development. The shift in the carbonation peaks in the region of 710–780 °C may indicate a densification of the cement matrix, slowing down the diffusion of CO_2_ [49,51]. It is worth noting that increasing the BPLB dosage to replace 30% of CEM III increases carbonation by almost two times. The analysis results in Figure 10a and Figure A1a lead to the conclusion that the by-products of BP from different productions exhibit good pozzolanic activity. This is evidenced by a comparison of the data for these modified cement pastes with partial replacement of CEM III with FA and SF (Figure A1c). It is important to note that at 30% FA, the strength decreases due to the excessive replacement of CEM III, which is likely a dilution effect. A similar situation can occur when replacing cement with 15% SF; however, due to its SiO_2_ content exceeding 90%, the strength of the samples remains high. This is presumably because of a significantly greater formation of dense C-S-H phases, as seen in Figure A1c.

Based on the data in Figure 10b for the modified compositions using CEM I, a similar trend of increased activity in the formation of denser structured C-(A)-S-H phases is observed with a reduction in BPL fineness. A significant increase in pozzolanic activity is noted at 30% replacement of CEM I with BPL; however, this did not lead to an increase in strength (Figure 7b,d). The likely reason for this is the uneven dispersion of structural elements throughout the material volume and the consequent increase in porosity [78]. Compositions with 15% BPL of different fineness show lower pozzolanic activity; however, due to a better distribution of hydrate phases, they possess higher strength (Figure 7b,d). The effect of reduced grinding fineness is also maintained during the dehydration of C-(A)-S-H phases when using BPLB (Figure A1b). Meanwhile, the highest portlandite consumption is recorded at 30% replacement with BPLB of 32 µm fineness. But due to a possible dilution effect, the highest strength is observed at 15% replacement with BPLB of the same fineness (Figure 7b,d). The behavior of FA and SF in systems with CEM I (Figure A1d) is nearly identical to that observed in systems incorporating CEM III. This correlation is consistent with the compressive strength results obtained for the studied blended cement mortars. Furthermore, the carbonation degree of all CEM I-based compositions remains low, irrespective of the dosage or fineness of the BP additive.

The aforementioned results lead to the conclusion that increasing the specific surface area of BP by reducing its particle size exerts a positive influence on its reactivity. The chemical composition of the BP, particularly its SiO_2_ content as the primary pozzolanic component, is also a critical factor. This finding suggests that intensive grinding, as required for the BLB additive to achieve high strength composites, may be unnecessary.

### 3.6. SEM

**Figure 11:**
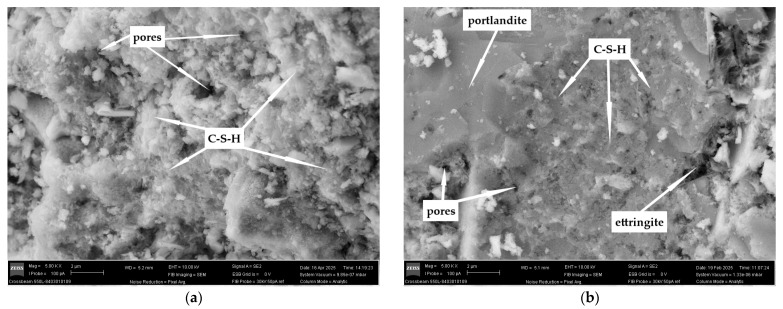
SEM photos (5000×) of the studied cement mortars based on CEM III (**a**), and based on CEM I (**b**).

**Figure A2:**
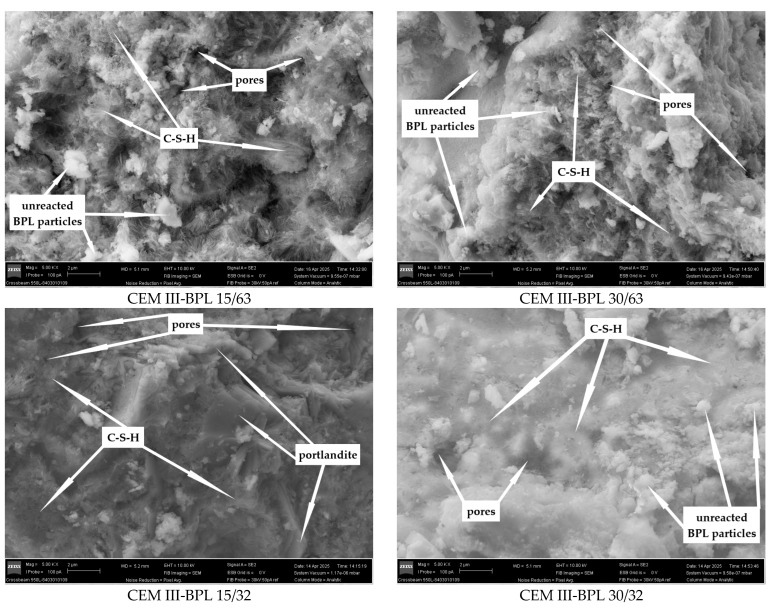

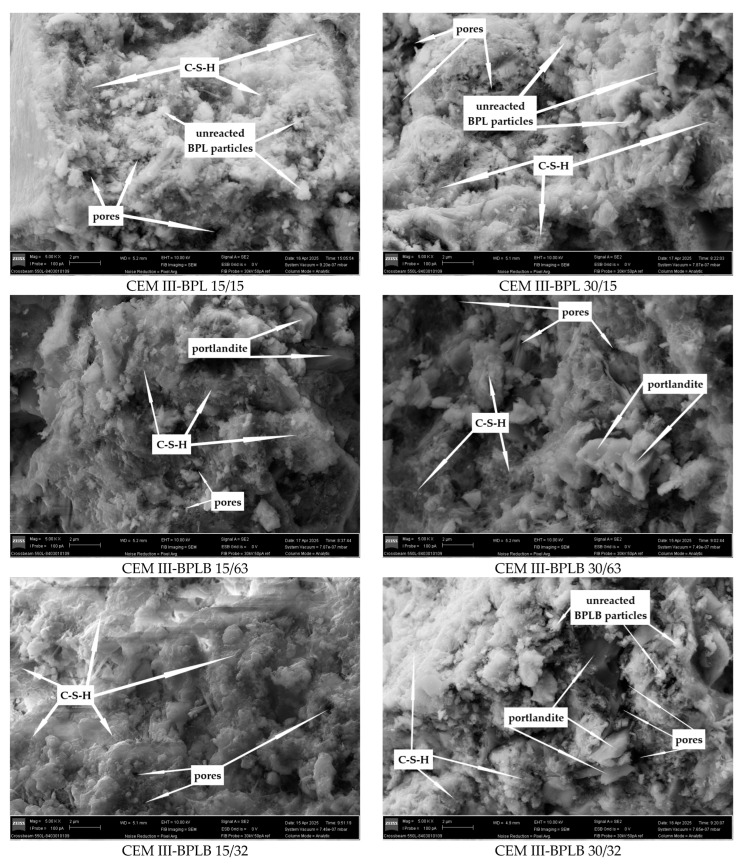

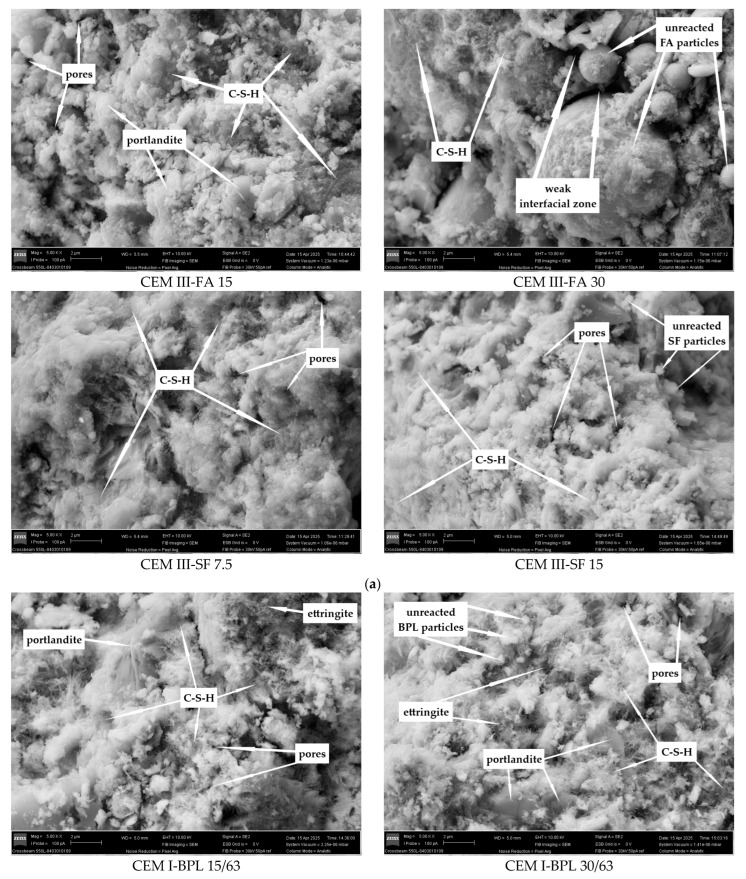

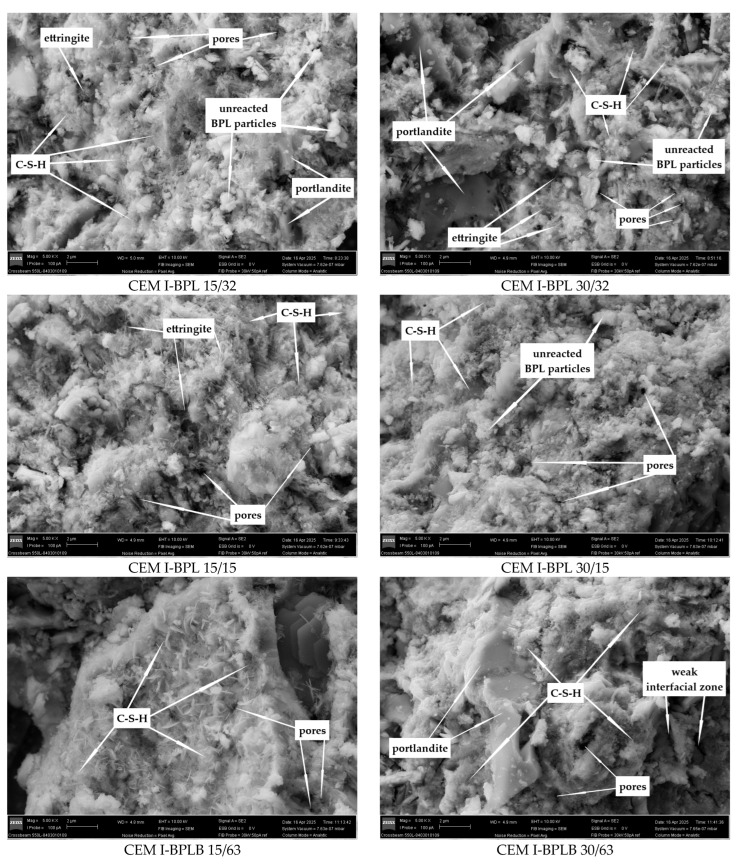

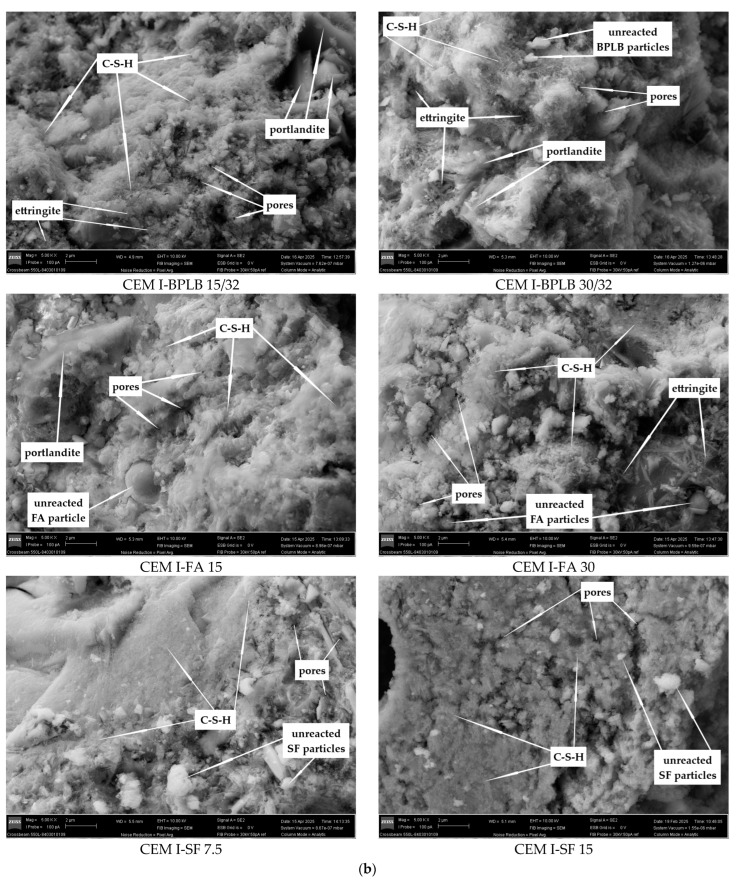
SEM photos (5000×) of the studied blended cement mortars based on CEM III (**a**), and based on CEM I (**b**).

The results of the SEM for the blended cement mortar compositions based on CEM III are presented in Figure 11a and Figure A2a, and those based on CEM I are shown in Figure 11b and Figure A2b.

Microstructural analysis of hydrate phase formation (Figure 11a and Figure A2a) corresponds well with previous TGA/DTG data of blended cement compositions based on CEM III. The revealed SEM images of modified compositions with 15% replacement of CEM III by secondary materials BPL and BPLB demonstrate a denser and more homogeneous microstructure compared to the 30% replacement level. In turn, a reduction in particle size enhances this effect in each case. Despite the near-complete absence of portlandite at 30% cement replacement with BPL and BPLB of different fineness, the formation of agglomerates of unreacted particles is clearly observed. This correlates with the TGA/DTG results shown in Figure 10a and Figure A1a. Such accumulations degrade the cement matrix and cause a microstructural imbalance of hydrate phases, which in turn leads to reduced strength (Figure 7a,c). The use of 15% FA is more advisable; 30% FA replacement not only reduces C-S-H quantity but also causes significant microstructural degradation due to poor bonding between the matrix and unreacted FA particles. Therefore, the effectiveness of modifying slag cement systems with SF is also clearly correlated with the TGA/DTG data (Figure A1c). Additionally, increased agglomeration of unreacted SF particles at the 15% dosage is confirmed.

Assessment of microstructural changes in blended cement pastes based on CEM I was performed using SEM images in Figure 11b and Figure A2b. The main distinguishing feature of the microstructure in compositions utilizing CEM I is the presence of ettringite compared to those with CEM III. This is explained by the differing hydration chemistry of ordinary Portland cements with high clinker content and slag-containing cements [9,79]. It is important to note that increased dosages of the by-products BPL and BPLB up to 30% significantly reduce the amount of ettringite, while a decrease in the dispersity of the additives has a less pronounced effect on the reduction in this hydrate phase’s concentration. The underlying mechanism involves a dilution of SO_3_ and an increase in Al_2_O_3_ reactivity, which accelerates the transformation of ettringite to AFm. In other respects, the effect of using different types of BP for replacing CEM I is similar to the replacement of CEM III. The optimal dosage of BPL and BPLB can be considered 15%, as at 30% replacement, numerous powder agglomerates form, resulting in a heterogeneous microstructure with significant defects that reduce strength (Figure 7b,d). The use of 15% FA promotes the development of a homogeneous, dense cement matrix with uniform distribution of hydrate phases compared to the 30% dosage. When using 7.5% SF, an ultra-dense microstructure forms, while increasing the dosage to 15% leads to a dilution effect due to agglomeration of unhydrated particles, explaining the slight reduction in strength (Figure 7b,d).

### 3.7. EDX

The results of the elemental analysis of the hydrated blended cement compositions are presented in Table 4 (mean values based on three points from different spectra). The obtained composition data in weight percent (wt.%) were recalculated into molar ratios (based on atomic fractions) for the quantitative assessment of the phase chemistry.

For EDX, samples were prepared using standard polishing methods and coated with a thin layer of carbon in a vacuum chamber to ensure electrical conductivity. The presence of the carbon coating and possible carbonization of the sample surfaces during storage contributed to the carbon signal in the EDX data. The main focus was on the comparative analysis of the elemental ratios within the hydrated phases (Ca/Si, Al/Si), as the contribution of the carbon coating to these values is systematic and identical for all samples.

**Figure 12:**
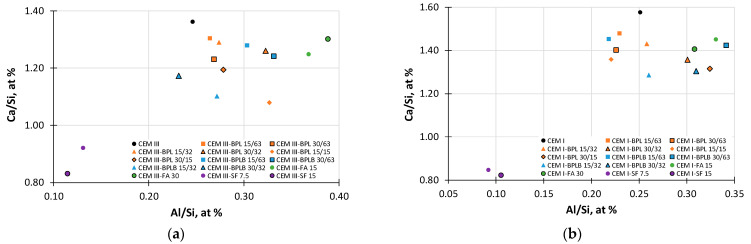
Phase cluster diagram Ca/Si-Al/Si for blended cement mortars based on CEM III (**a**), and based on CEM I (**b**).

Based on the data from Table 4, Ca/Si-Al/Si distribution diagrams (Figure 12) were plotted for the investigated blended cement mortars.

Analysis of the data in Figure 12 reveals clusters of points corresponding to the main phases present in the hydrated cement systems. The majority of data points for the CEM III-based compositions (Figure 12a) are concentrated in the region characteristic of C-(A)-S-H gel (a decrease in the Ca/Si ratio to the range of 1.08–1.2, accompanied by a simultaneous increase in Al/Si to 0.25–0.3) [80,81]. For compositions with an increased dosage of both types of BP, an elevated aluminum content (Al/Si > 0.32) is observed, indicating the presence of unreacted BP particles, which is confirmed by SEM (Figure A2a). An analogous trend is present in the compositions with FA, which contain an even greater amount of aluminum (Al/Si > 0.37). This indicates the presence of unreacted FA particles, as visible in the SEM images (Figure A2a), and is also explained by the chemical composition of this mineral additive. Clusters with the lowest Ca/Si values (<0.9) and Al/Si values (<0.13) are clearly visible, which is characteristic of cement systems modified with SF [82]. Thus, the Ca/Si-Al/Si diagram in Figure 12a clearly demonstrates that the replacement of CEM III with the BP waste by-products leads to a significant modification of the cement paste microstructure. This involves a reduction in the C-S-H gel basicity (pozzolanic reaction), its aluminization, and the subsequent formation of C-A-S-H.

Examining of the microstructural features of the CEM I-based compositions with BP (Figure 12b), a reduction in the basicity of the C-S-H phase is also observed (the Ca/Si ratio is in the range of 1.3–1.45), confirming the pozzolanic effect of the BP mineral additives. However, it is noteworthy that the compositions incorporating BPL are not as saturated with aluminum (Al/Si < 0.25) as the compositions with BPLB (Al/Si > 0.3), despite having an equal Al_2_O_3_ content in their chemical composition. The correlation of data with the SEM photos (Figure A2b) may indicate less reactive Al_2_O_3_ in BPL, which is supported by the presence of a larger volume of unreacted BPL particles compared to compositions containing BPLB. The behavior of SF and FA in CEM I-based compositions exhibits a similar pattern of microstructure cluster formation as for CEM III-based compositions.

## 4. Conclusions

In summary, the analysis of experimental data on the modification of blended cement composite using different cement types and brick production by-products of various origins leads to the following conclusions:

- Increasing the dosage of cement replacement with brick powder, regardless of cement type, raises the water demand of cement pastes and consequently reduces the workability of cement–sand mortars based on them;
- The setting times of cement pastes with both cement types decrease with reduced brick powder fineness. Despite nearly equal Al_2_O_3_ content, the amount of amorphous SiO_2_ in the powder plays a key role;
- The highest strength values can be achieved by replacing slag cement or ordinary fast-hardening cement with up to 15% BPL powder, fineness 15 μm or up to 15% BPLB powder, fineness 32 μm. This is attributed to the chemical composition of the by-products used. Thus, it can be concluded that a SiO_2_ deficit exceeding 5% in brick powder cannot be compensated by further grinding;
- Microstructural analysis using TGA/DTG, SEM, and EDX methods detailed the degree of hydrate phase formation after modifying blended cement composites. Specifically, the pozzolanic activity of the two brick powder types was confirmed, and the features of its chemical composition for the formation of low-basicity C-A-S-H gel in blended cement systems were established. The results demonstrate a strong correlation with strength indicators and the strength activity index.

The above findings indicate that brick production by-products with specific fineness and chemical composition can serve as an effective alternative to conventional SCMs—such as scarce fly ash and more expensive silica fume—on the German market. Integrating secondary products into production cycles represents a key element of the transition to a circular economy in the construction industry, while significantly reducing the carbon footprint.

A promising direction for future research is a comprehensive assessment of the durability of modified cement composites with the addition of brick powder. Key areas of interest include investigating shrinkage, resistance to carbonation, sulfate attack, and performance under cyclic thermal–humidity exposures.

## Figures and Tables

**Table 1:** Chemical composition of the used Portland cements and mineral additives based on XRF-analysis [54].

Oxide, %	CEM III	CEM I	BPL	BPLB	FA	SF
SiO_2_	27.04	20.41	56.68	63.06	51.71	97.14
CaO	52.35	62.14	8.33	6.56	4.25	0.30
Al_2_O_3_	8.71	5.63	16.87	16.71	25.61	0.15
Fe_2_O_3_	1.47	2.39	6.30	5.01	8.09	0.05
TiO_2_	0.52	0.31	0.89	0.76	1.23	-
MnO	0.12	0.06	0.09	0.08	0.07	-
MgO	4.05	1.60	5.26	2.32	1.95	0.15
K_2_O	0.64	0.77	3.56	3.47	2.37	0.22
Na_2_O	0.15	0.14	0.59	0.13	0.60	0.12
SO_3_	4.06	3.19	0.7	0.63	0.17	-
P_2_O_5_	0.06	0.11	0.18	0.24	0.48	0.03
LOI	1.09	3.08	0.95	1.07	3.15	1.18

**Table 2:** Mixing proportions of blended cement compositions.

Mixture ID *	CEM III, g	CEM I, g	BPL, g	BPLB, g	FA, g	SF, g	Sand, g	Water, g
CEM III	450	-	-	-	-	-	1350	225
CEM III-BPL 15/63	382.5	-	67.5	-	-	-		
CEM III-BPL 30/63	315	-	135	-	-	-		
CEM III-BPL 15/32	382.5	-	67.5	-	-	-		
CEM III-BPL 30/32	315	-	135	-	-	-		
CEM III-BPL 15/15	382.5	-	67.5	-	-	-		
CEM III-BPL 30/15	315	-	135	-	-	-		
CEM III-BPLB 15/63	382.5	-	-	67.5	-	-		
CEM III-BPLB 30/63	315	-	-	135	-	-		
CEM III-BPLB 15/32	382.5	-	-	67.5	-	-		
CEM III-BPLB 30/32	315	-	-	135	-	-		
CEM III-FA 15	382.5	-	-	-	67.5	-		
CEM III-FA 30	315	-	-	-	135	-		
CEM III-SF 7.5	416.25	-	-	-	-	33.75		
CEM III-SF 15	382.5	-	-	-	-	67.5		
CEM I	-	450	-	-	-	-		
CEM I-BPL 15/63	-	382.5	67.5	-	-	-		
CEM I-BPL 30/63	-	315	135	-	-	-		
CEM I-BPL 15/32	-	382.5	67.5	-	-	-		
CEM I-BPL 30/32	-	315	135	-	-	-		
CEM I-BPL 15/15	-	382.5	67.5	-	-	-		
CEM I-BPL 30/15	-	315	135	-	-	-		
CEM I-BPLB 15/63	-	382.5	-	67.5	-	-		
CEM I-BPLB 30/63	-	315	-	135	-	-		
CEM I-BPLB 15/32	-	382.5	-	67.5	-	-		
CEM I-BPLB 30/32	-	315	-	135	-	-		
CEM I-FA 15	-	382.5	-	-	67.5	-		
CEM I-FA 30	-	315	-	-	135	-		
CEM I-SF 7.5	-	416.25	-	-	-	33.75		
CEM I-SF 15	-	382.5	-	-	-	67.5		

* The mixture ID encodes cement type, additive type, additive content (%), additive fineness (for brick powder).

**Table 3:** Water absorption of used mineral additives, wt.%.

BPL 63 µm	BPL 32 µm	BPL 15 µm	BPLB 63 µm	BPLB 32 µm	FA	SF
55.1	82.8	96.9	40.4	58.8	48.5	217.8

**Table 4:** EDX data of the investigated compositions (at. %).

Mixture ID	O, %	Ca, %	Si, %	Al, %	Mg, %	Fe, %	S, %	K, %	C, %
CEM III	56.01	12.41	9.10	2.24	1.16	0.32	0.75	0.57	17.43
CEM III-BPL 15/63	54.04	13.10	10.04	2.65	1.34	0.44	0.76	0.26	17.35
CEM III-BPL 30/63	55.55	11.29	9.16	2.46	1.98	0.68	0.56	0.57	17.74
CEM III-BPL 15/32	54.48	12.36	9.57	2.62	1.83	0.36	0.88	0.21	17.68
CEM III-BPL 30/32	55.71	11.54	9.16	2.96	1.39	0.39	0.62	0.31	17.93
CEM III-BPL 15/15	55.97	10.47	9.70	3.17	1.96	0.43	0.81	0.25	17.23
CEM III-BPL 30/15	54.18	12.34	10.33	2.88	1.26	0.77	0.45	0.26	17.53
CEM III-BPLB 15/63	57.07	11.61	9.07	2.75	0.83	0.47	0.88	0.62	16.70
CEM III-BPLB 30/63	53.95	11.77	9.48	3.14	0.75	0.79	0.69	0.46	18.97
CEM III-BPLB 15/32	55.60	10.84	9.84	2.67	1.07	0.65	0.87	0.61	17.84
CEM III-BPLB 30/32	54.48	12.16	10.37	2.40	0.50	0.72	0.82	0.21	18.35
CEM III-FA 15	55.52	12.02	9.62	3.54	0.67	0.91	0.38	0.26	17.09
CEM III-FA 30	54.12	12.74	9.79	3.80	0.51	0.77	0.32	0.37	17.59
CEM III-SF 7.5	54.73	11.52	12.51	1.64	0.99	0.25	0.38	0.26	17.72
CEM III-SF 15	56.47	10.70	12.86	1.47	0.65	0.18	0.43	0.20	17.02
CEM I	57.79	13.26	8.41	2.11	1.00	0.77	0.64	0.26	15.77
CEM I-BPL 15/63	57.34	12.72	8.60	1.97	1.01	1.32	0.64	0.21	16.19
CEM I-BPL 30/63	56.31	13.30	9.49	2.14	1.19	0.85	0.71	0.37	15.65
CEM I-BPL 15/32	56.61	12.21	8.53	2.20	1.18	1.47	0.77	0.31	16.71
CEM I-BPL 30/32	55.48	12.74	9.38	2.82	0.85	1.14	0.71	0.42	16.46
CEM I-BPL 15/15	54.95	13.33	9.81	2.17	0.94	1.53	0.72	0.37	16.17
CEM I-BPL 30/15	57.50	11.62	8.84	2.87	0.92	1.17	0.70	0.47	15.92
CEM I-BPLB 15/63	56.23	13.20	9.09	1.98	1.19	1.14	0.64	0.26	16.28
CEM I-BPLB 30/63	54.96	13.11	9.20	3.14	0.60	1.22	0.65	0.42	16.70
CEM I-BPLB 15/32	56.53	11.97	9.30	2.42	0.84	1.39	0.64	0.26	16.65
CEM I-BPLB 30/32	56.20	12.49	9.57	2.97	0.59	1.07	0.90	0.16	16.06
CEM I-FA 15	55.45	13.53	9.32	3.08	0.68	1.04	0.78	0.21	15.91
CEM I-FA 30	56.01	12.87	9.14	2.82	0.59	1.07	0.71	0.37	16.42
CEM I-SF 7.5	55.66	11.03	13.01	1.20	0.83	0.65	0.44	0.21	16.98
CEM I-SF 15	54.98	11.20	13.59	1.43	0.75	0.55	0.51	0.26	16.74

## Data Availability

The original contributions presented in this study are included in the article. Further inquiries can be directed to the corresponding author.
